# Brivanib in combination with Notch3 silencing shows potent activity in tumour models

**DOI:** 10.1038/s41416-018-0375-4

**Published:** 2019-02-15

**Authors:** Catia Giovannini, Anna Maria Salzano, Michele Baglioni, Monica Vitale, Andrea Scaloni, Nicola Zambrano, Ferdinando Antonio Giannone, Francesco Vasuri, Antonia D’Errico, Gianluca Svegliati Baroni, Luigi Bolondi, Laura Gramantieri

**Affiliations:** 1 Center for Applied Biomedical Research (CRBA), S.Orsola-Malpighi University Hospital, Bologna, Italy; 2 Department of Medical and Surgical Sciences, University of Bologna, Bologna, Italy; 3 Proteomics & Mass Spectrometry Laboratory, ISPAAM, National Research Council, 80147 Napoli, Italy; 4 Dipartimento di Medicina Molecolare e Biotecnologie Mediche, Università degli Studi di Napoli Federico II, Napoli, Italy; 5 CEINGE Biotecnologie Avanzate S.C.aR.L, Napoli, Italy; 6 Pathology Unit, St. Orsola-Malpighi University Hospital, 40138 Bologna, Italy; 7 Polytechnical University of Marche, Ancona, Italy

**Keywords:** Cancer therapeutic resistance, Cancer therapy

## Abstract

**Background:**

Sorafenib is the first targeted agent proven to improve survival of patients with advanced hepatocellular carcinoma (HCC) and it has been used in first line treatments with heterogeneous response across patients. Most of the promising agents evaluated in first-line or second-line phase III trials for HCC failed to improve patient survival. The absence of molecular characterisation, including the identification of pathways driving resistance might be responsible for these disappointing results.

**Methods:**

2D DIGE and MS analyses were used to reveal proteomic signatures resulting from Notch3 inhibition in HepG2 cells, combined with brivanib treatment. The therapeutic potential of Notch3 inhibition combined with brivanib treatment was also demonstrated in a rat model of HCC and in cell lines derived from different human cancers.

**Results:**

Using a proteomic approach, we have shown that Notch3 is strongly involved in brivanib resistance through a p53-dependent regulation of enzymes of the tricarboxylic acid (TCA), both in vitro and in vivo.

**Conclusion:**

We have demonstrated that regulation of the TCA cycle is a common mechanism in different human cancers, suggesting that Notch3 inhibitors combined with brivanib treatment may represent a strong formulation for the treatment of HCC as well as Notch3-driven cancers.

## Background

Hepatocellular carcinoma (HCC) is the third leading cause of cancer mortality worldwide, with an increased incidence throughout the world.^1^ Although the introduction of screening programmes among high-risk populations leads to earlier diagnosis, the majority of patients presents with intermediate or advanced-stage disease. In this setting, only two treatments demonstrated survival advantages. Patients at an intermediate stage benefit from chemoembolisation, while sorafenib remains the only approved systemic drug for advanced stages.^2^ Many other molecular therapies have been explored in phase III clinical trials including those based on treatment with sunitinib, erlotinib, linifanib, lenvatinib, and brivanib. None of these drugs has shown positive results in the first-line setting.

Brivanib is a selective dual inhibitor of FGF and VEGF signalling that demonstrated antitumour activity in xenograft HCC models.^3^ In a phase II study, brivanib demonstrated promising antitumour activity as first line therapy in patients with advanced HCC.^4^ However, brivanib did not meet its primary end-point of overall survival (OS) non-inferiority versus sorafenib in the phase III BRISK-FL study.^5^ Reasons for failure should include liver toxicity, flaws in trials design and lack of understanding of critical drivers of tumour progression.^6^ For example, specific pathways are more often activated in some populations and this should be taken into account during trial design.^7^ In line with this observation, the clinical benefits of sorafenib treatment in HCC seem to differ between countries.^8^ The majority of patients in the phase III BRISK-FL study (60%) were from the Asia-Pacific region, where Notch pathway activation seems to be more relevant.^7^

In this perspective, we already assessed whether the Notch3 expression, which is strongly deregulated in HCC, is responsible for the resistance to brivanib.^9–11^ By using a rat model of HCC, we found in this study that Notch3 depletion combined with brivanib treatment exerts a substantial antitumour effect in vivo. Though a proteomic approach, we have highlighted the differential protein representation profile of control and Notch3-depleted cells exposed to brivanib. We have found that several enzymes of the tricarboxylic acid (TCA) cycle are differentially represented in Notch3-depleted cells and are functionally associated with brivanib resistance. We have also demonstrated here that Notch3 regulation of the TCA enzymes is p53 dependent. Finally, we have proved that this mechanism can be extended to other human cancer cells, thus suggesting that Notch3 inhibition holds a promise as a general strategy to improve brivanib effects.

## Material and methods

### Rat model and treatments

Sixty-five wistar rats were obtained from Harlan Italy (Udine, Italy), and were housed in an animal facility at Sant’Orsola-Malpighi Hospital (Bologna, Italy). Animals were maintained at 20–22 °C and were fed with a standard pellet diet ad libitum. The protocols of the experiments were approved by the Ethical Committee of the University of Bologna in accordance with European legislation. HCCs were induced by diethylnitrosamine (DENA). Protocols concerning HCC induction and Notch3 silencing were previously described.^12^ At the end of DENA treatment the animals were divided as follows: (1) DENA group; (2) siRNAN3 group; (3) DENA combined with brivanib; and (4) siRNAN3 combined with brivanib. Rat allocation in each group was performed by matching cases according to number and sise of nodules identified at serial ultrasound examinations, as well as for animal weight. Groups 3 and 4 rats were treated with 60 mg/kg of brivanib, which was dissolved in 6% hydroxypropyl cellulose (Sigma Chemical Company, St. Louis, MO). A fresh solution was made every day, and rats received treatment by oral gavages for 15 consecutive days.

### Cell lines and gene silencing by retroviral transduction of shRNAs

Human hepatocarcinoma cell lines HepG2, Hep3B, Huh7, and MCF7 were obtained from American Type Culture Collection (ATCC, Rockville, MD, USA), and were maintained according to ATCC instructions. TFK1, Huh28, and MDA-MB-468 cells were maintained in RPMI. Notch3 knock-down (KD) was obtained as previously described (Giovannini C., 2009). Since different Notch3-specific shRNAs were equally effective in our previous study,^13^ we performed the experiments by selecting single shRNAs. Cells harbouring a pSuper.puro provirus expressing a GL2 luciferase-specific shRNA were used as negative control.

### Brivanib and FACS analyses

Brivanib was obtained by Bristol-Meyers-Squibb. Stably infected cell populations of HepG2, Hep3B Huh7, MCF7, TFK1, Huh28 and MDA-MB-468 cells were seeded into six-well dishes and allowed to attach for 24 h before treatments with brivanib. Cell cycle analysis was performed as previously described after 48 h of brivanib exposure.^14^ Apoptosis was revealed by Annexin V-FITC (Bender Medsystems, Vienna, Austria) staining via FACS after 72 h of brivanib treatment.

### 2D-DIGE analysis

Protein extracts from three biological replicates of brivanib-treated and untreated HepG2 and HepG2 shN3 cells were obtained by lysis in 50 mM Tris–HCl, 150 mM NaCl, 0.5% Triton X-100, 50 mM NaF, 1 mM Na_3_VO_4_, 1 mM EDTA pH 8.0, 5 mM sodium pyrophosphate, 10 mM β-glycerophosphate and a mixture of protease inhibitors (Sigma Aldrich). Lysates were clarified by centrifugation at 12,000*g* for 30 min, at 4 °C. Proteins were precipitated in acetone/methanol (9:1, v:v) for 16 h, at −20 °C, and recovered by centrifugation at 16,000*g* for 30 min, at 4 °C. They were then dissolved in 7 M urea, 2 M thiourea, 4% CHAPS, 30 mM Tris–HCl; protein concentration was determined by using the Bradford method (Bio-Rad, Hercules CA, USA). Before labelling, the pH of the samples was adjusted to pH 8.5. Labelling reactions were performed in a 10 μL volume with 50 μg of the protein lysates, in the presence of 400 pmol of Cy2-dye, Cy3-dye, or Cy5-dye (minimal labelling dyes, GE Healthcare, Milan, Italy), by implementation of a dye-swapping strategy. Cell extracts were labelled with Cy3 or Cy5 for 30 min, at 0 °C, in the dark, and chased with 1 mM lysine. Three sample mixtures made of appropriate Cy3-labelled and Cy5-labelled pairs and a Cy2-labelled control, were supplemented with 1% v/v IPG buffer, pH 3–10 NL (GE Healthcare), 1.4% v/v DeStreak reagent (GE Healthcare), and 0.2% w/v DTT to a final volume of 450 μL in 7 M urea, 2 M thiourea, and 4% CHAPS. The mixtures (150 μg of total protein content) were used for passive hydration of IPG gel strips (24 cm, pH 3–10 NL) for 16 h, at 20 °C. IEF was performed on an IPGphor II apparatus (GE Healthcare) up to 80,000 V/h, at 20 °C (current limit, 50 μA/strip). The strips were equilibrated in 6 M urea, 2% SDS, 20% glycerol, and 0.375 M Tris–HCl (pH 8.8), for 15 min, in the presence of 0.5% w/v DTT, and then in the presence of 4.5% w/v iodacetamide in the same buffer, for additional 15 min, the whole procedure being performed in the dark. The equilibrated IPG strips were finally transferred onto 12% polyacrylamide gels, within low-fluorescence glass plates (ETTAN-DALT 6 system, GE Healthcare). The second dimension SDS–PAGE was performed on a DALT II electrophoresis unit (GE Healthcare) at 1 W/gel for 16 h. Gels were scanned on a Typhoon 9400 variable mode imager (GE Healthcare), with the indicated excitation/emission wavelengths for Cy2 (488/520 nm), Cy3 (532/580 nm), and Cy5 (633/670 nm). Images were acquired in the ImageQuant software (GE Healthcare) and analysed by using the DeCyder 6.0 software (GE Healthcare). A DeCyder differential in-gel-analysis module was used for spot detection and pairwise comparison of each to the standard present in each gel. The DeCyder biological variation analysis module was then used to simultaneously match all of the protein-spot maps from the gels, and to calculate average abundance ratios and *p* values across the triplicate sets of samples (Student’s *t*-test). For preparative protein separations, 500 μg of unlabelled total proteins from pooled extracts were used for passive hydration of 24 cm strips for the first gel dimension (pH 3–10 NL IPG strips, GE Healthcare). The first-dimension and second-dimension runs were carried out as described above. After preparative 2-DE, gels were fixed and stained with Sypro Ruby fluorescent stain (Invitrogen-Life Technologies Italia, Monza, Italy). After spot matching with the master gel from the analytical step in the biological variation analysis module of DeCyder software, a pick list was generated for spot picking, that was performed automatically by a robotic picker (Ettan spot picker, GE Healthcare).

### Protein identification

Spots from preparative 2-DE were excised from gels, minced, and washed with water. Proteins were in-gel reduced, S-alkylated, and digested with trypsin, as previously reported. Protein digests were subjected to a desalting/concentration step on μZipTipC18 pipette tips (Millipore Corp., Bedford, MA, USA) and then analysed by nLC-ESI-LIT-MS/MS. To this purpose, a LTQ XL mass spectrometer (Thermo Finnigan, San Jose, CA, USA) was used, which was equipped with Proxeon nanospray source connected to an Easy-nanoLC (Proxeon, Odense, Denmark). Peptide mixtures were separated on an Easy C_18_ column (100 × 0.075 mm, 3 μm) (Proxeon, Odense, Denmark) using a linear gradient from 5% to 50% of acetonitrile in 0.1% formic acid, over 30 min, at a flow rate of 300 nL/min. Spectra were acquired in the range *m*/*z* 400–1800. Acquisition was controlled by a data-dependent product ion scanning procedure over the three most abundant ions, enabling dynamic exclusion (repeat count 2 and exclusion duration 1 min). The mass isolation window and collision energy were set to *m*/*z* 3% and 35%, respectively. MASCOT software package version 2.3.02 (Matrix Science, UK) was used to identify spots from an updated human non-redundant sequence database (UniProtKB 2014/07). The following parameters were used: trypsin as proteolytic enzyme, a missed cleavages maximum value of 1, Cys carbamidomethylation as fixed modification, pyroglutamate (peptide *N*-terminal Gln) and Met oxidation as variable modifications. Data were searched by using a mass tolerance value of 2 Da for precursor ion and 0.8 Da for MS/MS fragments. Candidate proteins with more than two significant peptides (*p* < 0.05) identified with an individual MASCOT score > 30, were further evaluated by the comparison with their calculated mass and pI values, using the experimental values obtained from 2-DE.

### SDS–PAGE and Western blotting analysis

Protein extraction and quantification were performed as previously described^.^^15^ Primary antibodies were as follows: anti-Notch3 polyclonal antibody (sc-5593, Santa Cruz Biotechnology, Santa Cruz, CA), anti-Aconitase 1 (Aco1) polyclonal antibody (Novus Biological, Abingodon, UK), anti-P53 monoclonal antibody (Clone DO-7, Dako, Denmark), anti-Mdh1 polyclonal antibody (Novus Biological), anti-Idh1 polyclonal antibody (LSBio, Seattle, USA) and anti-β-Actin monoclonal antibody (Clone AC-40, Sigma). Immunoreactivities were revealed with the EnVision dextran polymer visualisation system (Dako). Membranes were washed and incubated with ECL (Cyanagen, Bologna, Italy). Signal acquisition was done with Chemidoc scanner (BioRad, Hercules, CA), and signals were quantified using a specific densitometric software (Image Lab, BioRad) in absorbance units after light calibration with a reference autoradiography.

### Small interfering RNA transfections

Cells used in the study were seeded into six-well plates and transfected with 20 nM P53 (Invitrogen), ACO1, MDH1, IDH1, (IDT, Thief River Falls, MN, USA) or scrambled siRNA (NC) using Lipofectamine2000 (InVitogen). Transfection efficiencies were >90% as determined by co-transfection with a fluorescein-labelled siRNA (InVitrogen). Five hours were allowed to elapse before treatment with brivanib. Cells were collected at 5 and 72 h post-transfection, and protein representation was analysed by western blotting.

### Biochemical assay

Lactate release was determined in the medium of HepG2 cells by using a Lactate PAP Fluid Kit (Centronic GmbH, Wartenberg, Germany), following the manufacture’s instruction. Data were expressed as micromole/10^6^ cells and normalised to the number of cells present in the wells. The oxygen consumption rate was measured by using an oxygen Clark-type electrode and expressed as nanomole of O_2_/min/mg of protein.

### Immunohistochemistry (IHC)

The presence and localisation of Notch3 (Santa Cruz Biotecnology) in 60 CCA was immunohistochemically assessed on formalin-fixed, paraffin-embedded sections as previously described.^16^ Staining of sections was assessed on 10 consecutive ×20 magnification fields by two independent observers (L.G., C.G.) using a validated semi-quantitative scale where –, absence of staining; ±, occasional weak cholangiocytes staining; +, staining of >5% cholangiocytes ++, staining of 5–30% cholangiocytes and +++ staining on >30% cholangiocytes.

### Statistical analysis

Differences between groups were analysed using a double-sided Student's *t*-test. *P*-values <0.05 were considered statistically significant. Statistical analyses were performed using SPSS version 19.0 (IBM Corp., Armonk, NY).

## Results

### Notch3 silencing enhances the effect of brivanib in vivo

**Fig. 1:**
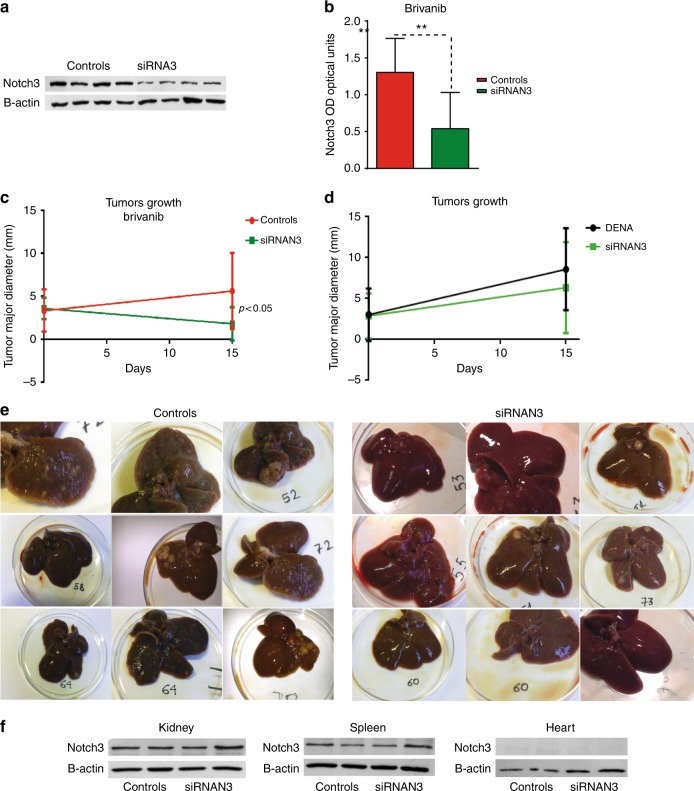
In vivo evidence of the role of Notch3 in brivanib resistance. **a** Representative Notch3 expression in rat HCC nodules detected by western blotting. Higher Notch3 expression was observed in controls, compared to Notch3-silenced rats (siRNAN3). All rats were treated with brivanib for 15 days. **b** Notch3 levels detected by western blotting were quantified and expressed as ratio with respect to housekeeping β-actin. **p < 0.01 (by two tailed Student’s *t*-test). **c** A difference in tumour growth was evident between controls vs. siRNAN3 after 15 days of brivanib treatment. *p* < 0.05 by two-tailed Student’s *t*-test. **d** Tumours growth analysis in DENA treated rats and in Notch3-depleted animals. **e** Macroscopic examination showing the upper and lower surfaces of representative cases of controls and siRNAN3 livers treated with brivanib for 15 days. Livers from rats treated by brivanib associated with siRNAN3 displayed a lower nodularity, as well as a lower number of tumours. **f** siRNAN3 does not affect Notch3 expression in Kidney, spleen and heart as evaluated by western blot

Recent studies have revealed the contribution of several specific signalling pathways in the acquisition of resistance to cancer therapy.^17^ Due to its role in maintaining the balance between cell proliferation and apoptosis, Notch signalling is involved in various aspects of cancer biology, from stemness maintenance to multidrug resistance.^18^ In HCC, Notch3 has been described to play a role in cell proliferation, apoptosis, as well as in multi-drug resistance.^13,19–21^ To assay whether Notch3 is involved in mediating resistance to brivanib in vivo, we used a model of rat HCC induced by DENA characterised by high Notch3 expression.^12^ Our experiments showed that by treating rat HCC with brivanib (60 mg/kg/day) for 15 days, tumour growth is significantly inhibited in Notch3-depleted HCCs than in control (Fig. 1a–c). On the contrary, no significant difference was observed in tumour growth between DENA-treated and Notch3-depleted animals, without brivanib (Fig. 1d). Macroscopic liver examination of rat livers treated with brivanib alone or in combination with Notch3 siRNAs showed a lower nodularity of liver parenchyma in rats treated with the combination. Moreover, brivanib plus Notch3 inhibitor treated rats displayed a significantly lower HCC nodules number, when compared with brivanib-treated animals (Fig. 1e). We did not observe any complication in terms of liver damage, body weight loss, or distress in siRNA rat model. Remarkably, we observed a specific Notch3 siRNA delivery to the liver probably avoiding major complications (Fig. 1e).

### Notch3 silencing enhances effect of brivanib in HCC cells

**Fig. 2:**
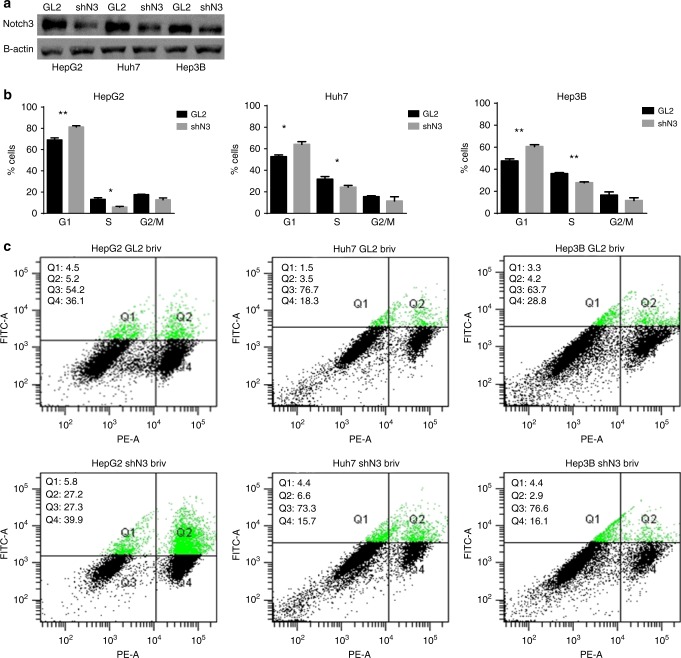
Effects of Notch3 silencing on brivanib response in vitro. **a** Efficacy of Notch3 silencing (shN3) was measured by western blotting in HepG2, Huh7 and Hep3B cells. **b** Cell cycle distribution of control (GL2) and Notch3-depleted (shN3) cells in response to 60 μM brivanib were quantified by FACS analysis after 48 h of treatment. Results are the mean of three independent experiments (±S.E.). *p < 0.05; **p < 0.01 values by two-tailed Student’s *t*-test. **c** After treatment with 60 μM brivanib for 72 h, HepG2, Huh7 and Hep3B cells were labelled with annexin V-FITC and propidium iodide. The distribution pattern of live and apoptotic cells was determined by FACS analysis. *x*-axis represents propidium staining (PE) and *y*-axis represents FITC staining. Data are representative of at least three independent experiments

The correlation between Notch3 expression and response to brivanib observed in vivo, suggested that the effect of brivanib on cells growth can be strengthened by Notch3 silencing (Fig. 2a). In agreement with this hypothesis, we showed that Notch3 ablation exacerbated the response to brivanib affecting proliferation of HepG2, Huh7 and Hep3B cells. Specifically, an increase of the G1 cell population was observed in Notch3-depleted cells (shN3) treated with brivanib, when compared with cells treated only with brivanib (Fig. 2b). We previously showed that Notch3 depletion does not affect rate of apoptosis of HCC cells. However, Notch3 ablation exacerbated the apoptotic response to doxorubicin.^13^ Accordingly, the mortality due to Notch3 silencing increased by 3.4-fold and 2.2-fold in HepG2 and Huh7 cells, respectively, after 72 h of exposure to brivanib, whereas no difference was observed in Hep3B, as revealed by Annexin V-FITC staining (Fig. 2c). These findings outline the functional relevance of Notch3 ablation in association with brivanib, which is however influenced by different cell contexts.

### Functional classification of the differential expression signature of shN3 cells treated with brivanib

**Fig. 3:**
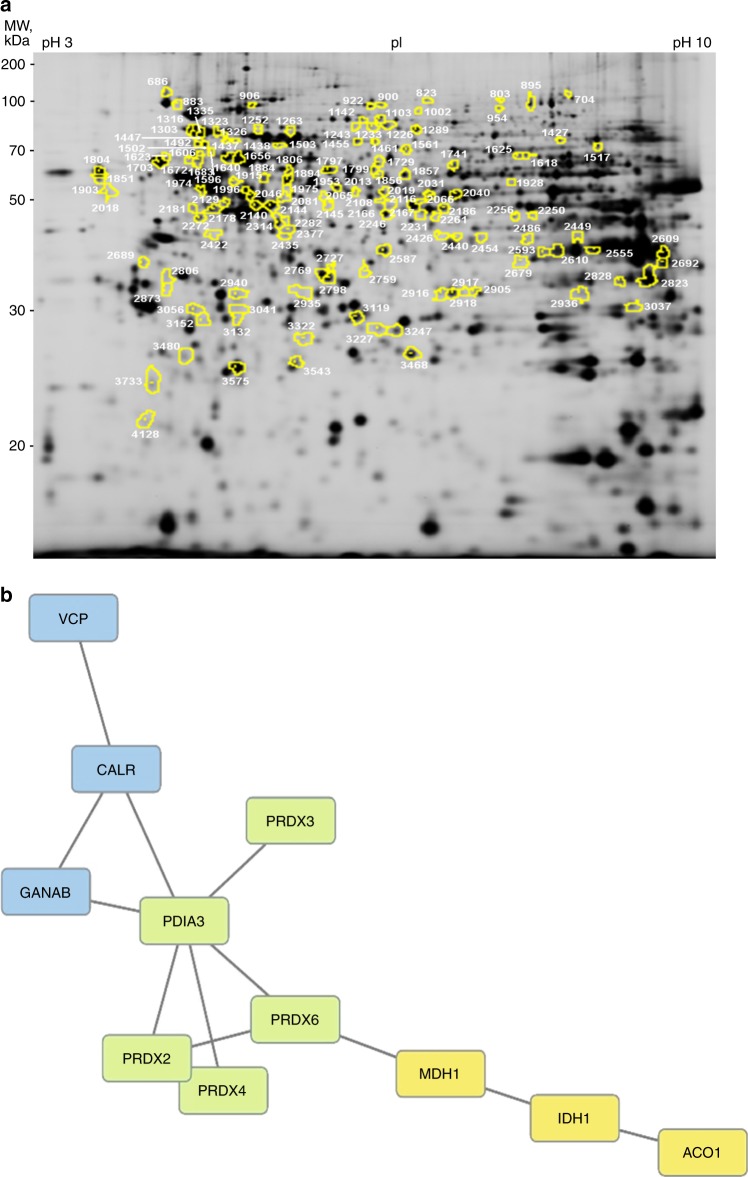
Proteomic analysis of HepG2-GL2 and HepG2-shN3 preventively treated with brivanib. **a** Analytical two-dimensional gel electrophoresis of HepG2 proteins. The figure reports the Cy2-labelled proteins on the scanned master gel. Preparative and analytical assays were performed as reported in the experimental section. Differentially represented spots are numbered in the gel image; they were picked automatically from a preparative gel for protein identification and analysed for their tryptic digests by nLC-ESI-LIT-MS/MS. Protein identification results are reported in Supplementary Table 1. **b** STRING analysis of differentially represented proteins identified by combined 2D-DIGE/nLC-ESI-LIT-MS/MS analysis. Protein acronyms are reported in capital letters

We then used a proteomic approach to investigate the mechanisms associated with the enhanced brivanib sensitivity observed in Notch3-depleted cells. To this aim, we analysed the proteomic profiles of HepG2-GL2 and HepG2-shN3 cells treated with brivanib for 72 h, taking advantage of two-dimensional differential in-gel electrophoresis (2D-DIGE) technology; these cells showed the highest difference in the Annexin V-FITC assay. Three biological replicates were used for this pourpose. A representative analytical 2D-DIGE gel is shown in Fig. 3a. Approximately 5492 protein spots were resolved within a pH 3–10 range and a 10–200 kDa molecular mass range. About 3774 valid spots were matched throughout the gels for DeCyder statistical analysis. Protein spots with an average abundance ratio ≥ 1.50 or ≤−1.50 (*P* ≤ 0.05) between HepG2-GL2 and HepG2-shN3 cells, both treated with brivanib for 72 h, were considered as differentially represented spots (DRSs). This analysis revealed 137 DRSs, of which 62 and 75 were over and down-represented in HepG2-shN3 cells, respectively, in comparison with HepG2-GL2 counterpart. DRSs were subjected to protein identification by nanoliquid chromatography coupled to electrospray-linear ion trap-tandem mass spectrometry (nLC-ESI-LIT-MS/MS) analysis and database searching of resulting mass spectrometric data. Reliable quantitative abundance data were obtained for 50 DRSs associated with a single-protein identification (Supplementary Tables 1 and  2), while the remaining ones corresponded to multiple comigrating protein species present in each gel spot. Accordingly, only the first ones were further considered for evaluating the proteomic variations specifically associated with Notch3 depletion in brivanib-treated cells; these 50 DRSs corresponded to 42 differentially represented proteins (DRPs).

In order to classify above-reported DRPs according to the biological processes in which they are involved, we used the GO-enrichment tool in the DAVID platform. The highest ranked processes affected by Notch3 silencing in HepG2 cells treated with brivanib are reported in Supplementary Table 3. The most significantly enriched term was “cell redox homoeostasis” (*P*-value 3.0E−5, Benjamini 1.0E−2) and contained five proteins, namely peroxiredoxin 2 (Prdx2), peroxiredoxin 3 (Prdx3), peroxiredoxin 4 (Prdx4), peroxiredoxin 6 (Prdx6) and protein disulphide isomerase family A member 3 (Pdia3). In order to define relationships occurring among the members of the proteomic signature here identified, a STRING interaction analysis was also performed. By setting the minimum required interaction score to 0.7 (high confidence), a unique major interaction network was evidenced (Fig. 3b). It involved the above-mentioned proteins related to cell redox homoeostasis, which were connected with two additional blocks of three proteins each. The first block contained proteins involved in the TCA cycle, namely aconitase 1 (Aco1), isocitrate dehydrogenase (Nadp+) cytosolic (Idh1) and malate dehydrogenase 1 (Mdh1), while the second one included proteins involved in the pathway of protein processing in the endoplasmic reticulum (Kegg Pathway hsa04141), namely calreticulin (Calr), neutral alpha-glucosidase AB (Ganab) and transitional endoplasmic reticulum ATPase (Vcp).

### Validation of the proteomic signature

**Fig. 4:**
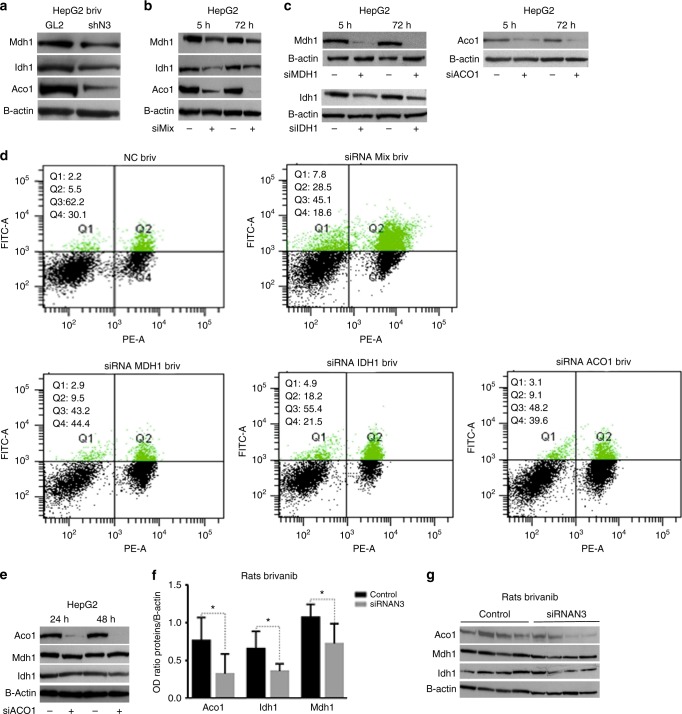
Enzymes of the TCA cycle are involved in brivanib resistance. **a** Western blotting analysis confirms a down-representation of Mdh1, Idh1 and Aco1 proteins in Notch3-depleted HepG2 cells compared to GL2 control cells, when both were exposed to 60 μM brivanib for 72 h. **b–d** HepG2 were transiently transfected with a pool of siRNAs (siMix) directed against Mdh1, Idh1, Aco1 or negative control (NC) for 5 and 72 h. Single gene silencing was also performed as showed in panel **c**. Five hours post-transfection, cells were treated with 60 μM brivanib for 72 h, and apoptosis was assessed by annexin V-FITC and propidium iodide. Data are representative of at least three independent experiments. **e** Aco1 silencing, the first enzyme involved in the TCA cycle, does not affect the representation of Mdh1 and Idh1, as evaluated by western blotting. **f** Aco1, Idh1 and Mdh1 protein representation was detected by western blotting in rat HCCs exposed to brivanib for 15 days; it is expressed as ratio with respect to β-actin housekeeping. *p < 0.05 (by two-tailed Student’s *t*-test). **g** Representative western blot analyses of Aco1, Mdh1 and Idh1 protein representation in rat HCCs treated either by brivanib alone or in combination with Notch3 inhibitors

Among the most enrichment terms, 2D-DIGE-based proteomic results outlined down-representation of metabolic enzymes and over-representation of proteins involved in cell redox homoeostasis in Notch3- depleted cells treated with brivanib, compared to negative control. Peroxiredoxins (Prdxs) are antioxidant enzymes emerging as very important in protecting cells against reactive oxygen species (ROS).^22^ Although their protective role in cardiovascular and neurological diseases is well established, their role in cancer is controversial.^23^ Considering the oncogenic role of Notch3 in HCC (9), we can assume that its silencing results in over-representation of proteins playing a role of tumour suppressor and down-representation of proteins with a more oncogenic function. In accordance with both our hypothesis and findings, several studies have already demonstrated a tumour suppressor function of prdxs in HCC.^24–26^ Given the ability of carcinogenic cells to reprogramme their metabolism towards aerobic glycolysis and the lack of studies especially on Aco1 and Mdh1 in HCC, we chose three proteins of the TCA cycle for validation of 2D-DIGE-based proteomic results. As shown in Fig. 4a, western blotting analysis of protein lysates from HepG2-GL2 and HepG2-shN3 cells exposed to brivanib for 72 h confirmed a down-representation of Mdh1, Idh1 and Aco1 in Notch3-depleted HepG2 cells. To determine whether Mdh1, Idh1 and Aco1 are major factors associated with the enhanced brivanib sensitivity in Notch3-depleted cells, we ablated endogenous levels of Mdh1, Idh1 and Aco1 by transient siRNA transfection of HepG2 cells either separately or simultaneously (Fig. 4b, c). Five hours post-transfection, cells were treated with 60 μM brivanib for 72 h. All single gene silencing significantly affected response to brivanib, when compared to scramble-transfected cells (Fig. 4d). Simultaneous down-regulation of Mdh1, Idh1 and Aco1 mRNA strongly exacerbated brivanib effect, as revealed by Annexin-V analysis. Cell death upon brivanib exposure increased 3.9-fold in cells simultaneously silenced for Mdh1, Idh1 and Aco1, compared to negative control (Fig. 4d). Considering that Notch3 depletion alone enhances brivanib sensitivity of about 3.4-fold in HepG2 cells (Fig. 1b), we can speculate that Mdh1, Idh1 and Aco1 expression strongly contribute to the increased brivanib sensitivity of Notch3-silenced cells. Since Aco1 is one of the first enzymes involved in the TCA cycle, we wondered whether its reduced representation could alter the expression of downstream enzymes because of reduced levels of substrates. Interestingly, Aco1 silencing did not affect the expression of the other enzymes analysed in this study, thus suggesting that Notch3 controls their expression independently (Fig. 4e and Supplementary Fig. 1A). To assess the function of the TCA cycle, lactic acid and O_2_ consumption were measured in HepG2-GL2 and HepG2-shN3 cells exposed to brivanib for 72 h and found that silenced cells are characterised by a lower respiration rate whereas no difference was observed in lactic acid production suggesting that Notch3 depleted cells do not switch to anaerobic respiration (Supplementary Fig.1B–C). To assay whether Notch3-mediated regulation of Mdh1, Idh1 and Aco1 protein expression really occurs in primary tumours, we examined protein abundance in Notch3-silenced and control rats after brivanib treatment. We found that Notch3-silenced tumours have significant lower levels of Aco1, Idh1 and Mdh1 than control counterparts (Fig. 4f, g). The correlation between in vivo and in vitro studies suggested that, by reducing Mdh1, Idh1 and Aco1 levels, Notch3 inhibition enhances sensitivity to brivanib treatment.

### P53 is required to enhance the cytotoxicity of brivanib

**Fig. 5:**
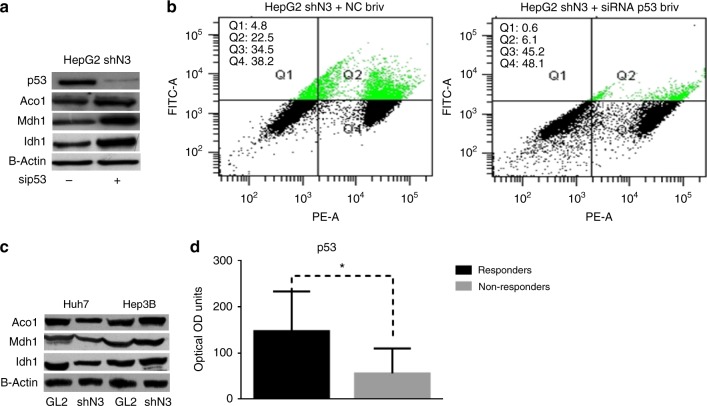
P53 is required to enhance the cytotoxicity of brivanib. **a** and **b** Endogenous p53 expression was ablated by transient siRNA transfection in Notch3-depleted HepG2 cells, and p53, Aco1, Mdh1 and Idh1 protein levels were evaluated by western blotting 5 h post-transfection. Cells were treated with 60 μM brivanib for 72 h, and apoptosis was assessed by annexin V-FITC and propidium iodide. Data are representative of three independent experiments. **c** Aco1, Mdh1 and Idh1 protein representation evaluated in Huh7 and Hep3B control cells (GL2) and in Notch3KD cells (shN3) by western blotting. **d** p53 protein representation evaluated by ELISA in HCC tissue of 20 rats treated with brivanib for 15 days and grouped accordingly to response to treatment

We previously showed that Notch3 knock-down results in the accumulation of p53 mediated by a dramatic decrease of cyclin G1 and sustained by MDM2 and miR221 axis both in vitro and in human HCC.^12^ Moreover, we demonstrated that p53 accumulation induced by Notch3 silencing enhances the apoptosis inducing effect of doxorubicin, while it has not effect on sorafenib response.^13,20^ To determine if higher p53 levels were functionally associated with the enhanced brivanib sensitivity of Notch3 KD HepG2 cells, we ablated endogenous p53 expression by transient siRNA transfection of Notch3 KD cells (Fig. 5a). Transfected cells were treated with brivanib (60 μg/ml) for 72 h. P53 depletion abolished the enhanced apoptotic reaction of Notch3-depleted cells, as revealed by Annexin-V in three independent experiments (Fig. 5b). Remarkably, Mdh1, Idh1 and Aco1 levels were significantly over-represented in p53-depleted cells (Fig. 5a). This evidence was further supported by the over-representation of Mdh1, Idh1 and Aco1 observed in Hep3B (p53-/-) Notch3-depleted cells upon brivanib exposure; the latter are resistant to brivanib as control cells (Fig. 5c). To assess to what extent our in vitro findings were representative of what occurs in vivo, p53 protein was assessed using ELISA in HCC rats after brivanib treatment. We found that p53 levels were significantly higher in rats responding to brivanib treatment than in non-responders (Fig. 5d). These findings suggest a pivotal role of p53 in the modulation of the TCA cycle-related enzymes in HCC cells treated with brivanib; this modulation might affect treatment efficacy.

### Notch3 is highly expressed in cholangiocarcinoma (CCA)

**Fig. 6:**
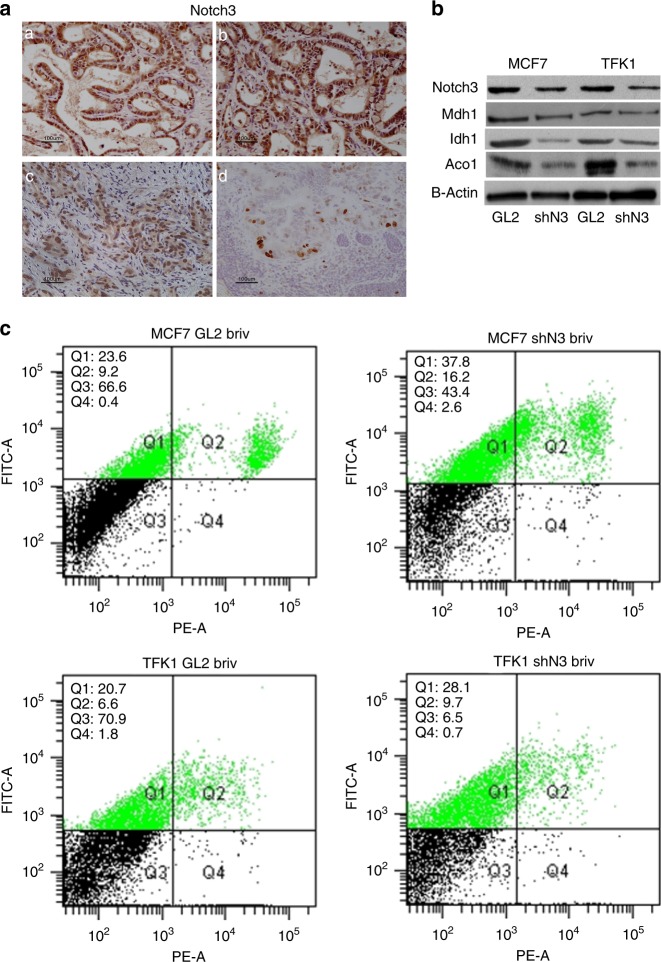
Notch3 confers resistance to brivanib in different tumour models. **a** Immunohistochemistry of Notch3 in four representative cases of CCA (**a–d**). Notch3 accumulation was evident in the nucleus. Scale bars = 100 μm. **b** Mdh1, Idh1 and Aco1 protein representation evaluated by western blotting in MCF7 and TFK1 negative control (GL2) and Notch3-silenced cells (shN3). **c** After treatment with 60 μM brivanib for 72 h, MCF7 and TFK1 cells were labelled with annexin V-FITC and propidium iodide. The distribution pattern of live and apoptotic cells was determined by FACS analysis

Notch3 expression has been poorly investigated with different results in CCA.^27,28^ Herein we analysed Notch3 expression in 60 CCA and in matched non-tumour tissues; we found that it was aberrantly expressed in 75% of patients in malignant ductules, with a nuclear localisation suggesting its functional role (Fig. 6a). In line with previous studies, Notch3 was also not detected in normal hepatocytes as well as in endothelial and inflammatory cells.^16,27^ Despite a high percentage of CCA displayed Notch3 up-regulation in tumour tissue, any specific correlation with tumour characteristics was identified (Table 1). These findings support further investigation on Notch3 inhibition associated with brivanib treatment in CCA.

**Table 1:** Characteristics of CCA patients

Gender (M/F)	46/14	
Age (median ± SD)	67.2 ± 7.9	
*Localisation: Intra/extrahepatic CCA*		
Intra	34/60 (56.7%)	NOTCH3 pos: 22
		NOTCH3 neg: 9
		intermediate: 3
Extra	26/60 (43.3%)	NOTCH3 pos: 16
		NOTCH3 neg: 6
		intermediate: 4
*Nodule size*		
<3 cm (%)	18/60 (30%)	NOTCH3 pos: 9
		NOTCH3 neg: 6
		intermediate: 3
>3 cm (%)	42/60 (70%)	NOTCH3 pos: 29
		NOTCH3 neg: 9
		intermediate: 4
*Grading*		
G1	3/60 (5%)	NOTCH3 pos: 1
		NOTCH3 neg: 1
		intermediate: 1
G2	32/60 (53.3%)	NOTCH3 pos: 18
		NOTCH3 neg: 10
		Intermediate: 4
G3	23/60 (38.3%)	NOTCH3 pos: 18
		NOTCH3 neg: 3
		Intermediate:2
*Epatocholangioca*	2/60 (3.3%)	NOTCH3 pos: 1
		NOTCH3 neg: 1
*Growth type*		
Mass forming	40 (66.7%)	NOTCH3 pos: 27
		NOTCH3 neg: 9
		Intermediate: 4
Peri-ductal infiltrating	18 (30%)	NOTCH3 pos: 10
		NOTCH3 neg: 5
		Intermediate: 3
Intra-ductal	2 (3.3%)	NOTCH3 pos: 1
		NOTCH3 neg: 1

NOTCH3 pos: >30% neoplastic nuclei

NOTCH3 neg: <5% neoplastic nuclei

Intermediate: Notch3 positivity in 5–30% neoplastic nuclei

### Notch3 silencing enhances brivanib activity in different tumour models

Aberrant activation of Notch3 signalling is linked to cancer cell seeding and development of breast cancer metastasis, in particular enhanced Notch3 expression may stand as a novel mechanism for promoting bone metastasis.^29,30^ Notch3-mediated signalling plays an important role in the proliferation of ErbB2-negative breast cancers^31^ and Notch3 signalling can drive an oncogenic programme in a subset of basal breast cancer.^32^ Notch3 was found to be expressed in breast cancer but not in normal breast tissues and an elevated Notch3 expression was found in patients with low Notch1 levels.^33^ A high Notch3 expression was also described in triple-negative breast cancers (TNBCs) where it is co-expressed with EGFR and its inactivation increases sensitivity to gefitinib.^34^ Based on our results on CCA and extensive literature on Notch3 expression and role in breast cancer,^32,35,36^ we investigated whether the enhanced brivanib activity due to Notch3 silencing observed in HCC can be extended to these tumours. To this aim, we analysed two different cell lines deriving from CCA (TFK1) and breast cancer (MCF7). As shown in Fig. 6b, Notch3 silencing in MCF7 and TFK1 cells results in Mdh1, Idh1 and Ido1 down-representation, as observed in HepG2 and Huh7 HCC cell lines, suggesting that Notch3 regulation of metabolic pathways is a common feature in different human cancer cells. However, enhanced brivanib sensitivity due to Notch3 ablation was more evident in MCF7 than in TFK1 cells (Fig. 6c) probably due to their different p53 status, as observed for HepG2 and Huh7 HCC cells. Indeed, TFK1 and Huh7 display p53 mutations, which might reduce p53 activity, thus affecting the extent of their sensitivity to brivanib treatment. Two additional TP53-mutated cell lines were thus assayed to explore the effects of brivanib plus Noth3 inhibition in this setting. In line with our hypothesis, the apoptosis inducing effect of brivanib was less evident in p53 mutated MDA-MB-468 cells compared to MCF7 cells upon Notch3 inhibition. Moreover, Notch3 ablation in Huh28 p53-mutated CCA cells have similar effects to TFK1 in response to brivanib as shown by Annexin-V staining (Supplementary Fig. 2A-B). These findings further support the hypothesis that a functional TP53 could be considered as a putative biomarkers for selection of cases which might take advantage from brivanib associated with Notch3 inhibition.

## Discussion

Cancer cells have metabolic alterations, allowing them to satisfy the metabolic needs necessary for the increased proliferation and malignancy. Because many altered metabolic features can be found in different types of human cancer, metabolism is now considered a hallmark of cancer.^37,38^ Recently, the enzymes of the TCA cycle, which are located in the mitochondrial matrix, have been extensively studied in the field of oncology. Indeed, a role of mitochondrial function in cancer therapeutic resistance has been demonstrated,^39^ suggesting that mitochondrial inhibitors may limit cancer progression. However, inhibition of metabolic enzymes could be systemically toxic because of their physiological functions in normal tissues.^40^ Normal proliferating cells, such as immune cells, display metabolic features resembling cancer cells; thus, metabolic inhibitors may interfere with the adaptive immune system. Identifying specific cancer proteins that regulate metabolic enzymes would be of crucial importance for tumour treatment. Notch3 is a transmembrane receptor widely expressed during embryonic development. Conversely, its expression in adult human tissues is limited to the central nervous system, to vascular smooth muscle cells^41^ and to very few subset of B cells.^42^ Thus, Notch3 aberrant expression in human cancers represents a promising target for therapeutic treatment.^11,16,43,44^ Herein, we have shown that Notch3 regulates Aco1, Mdh1 and Idh1, three important enzymes of the TCA cycle leading to brivanib resistance in HCC. Notably, our results were confirmed both in vitro and in vivo ruling out that nutrients used in culture identify activities that may be less important in vivo. Remarkably, heterogeneous results were here obtained from three HCC cell lines displaying different p53 status. Notch3 silencing combined with brivanib treatment affected the cell cycle of all the analysed cell lines, whereas increased apoptosis was observed only in HepG2 and Huh7 cells, thus suggesting a possible role of p53 in brivanib-induced apoptosis. This experimental result is in accordance with the reduced time to progression observed in the phase II trial. However, the lack or reduced apoptosis observed in the absence of functional p53 would explain the lack of brivanib benefit on OS observed in the phase III BRISK-FL study. In line with this observation, a role of p53 in regulating mitochondrial metabolism, as well as increased p53 expression in Notch3-depleted cells were described.^13,45^ Furthermore, the combined silencing of Notch3 and p53 in HepG2 p53+/+ cells triggered the over-representation of Mdh1, Aco1 and Idh1 proteins and abrogated the enhanced apoptotic reaction of the Notch3-depleted cells, mimicking the effects observed in Hep3B TP53−/− cells. According to in vitro results, we found higher p53 levels in rats responding to brivanib treatment compared to non-responders. The majority of cancers, regardless of tissue origin, are characterised by disorders in mitochondrial metabolism.^46,47^ In this regard, it should be emphasised that success of imatinib (Gleevec) in managing BCR-ABL leukaemia cells is dependent on their ability to target signalling pathways linked to glucose metabolism.^48^ Thus, we investigated whether the enhanced brivanib activity associated with reduced levels of mitochondrial enzymes, as resulting from Notch3 silencing, can occur also in other human cancer cells. Across other cancer cell lines tested here, brivanib-induced apoptosis of CCA cells and breast cancer cells subsequently to Notch3 silencing. Interestingly, we found that Notch3 ablation reduced Mdh1, Aco1 and Idh1 representation in both MCF7 and TFK1 cells. Taken together, our results suggest that patient’s selection based on molecular signatures including Notch3 and p53 analysis can identify which patients may benefit from brivanib treatment. Indeed, in the context of functional p53, we can hypothesise that Notch3 is a specific positive brivanib resistance factor acting, at least in part, on the enzymes of the TCA cycle. Our results provide a strong rationale for brivanib evaluation combined with Notch3 inhibitors in the treatment Notch3-driven cancers. This study highlights the possibility of using brivanib for the treatment of different human tumours, especially for molecularly defined subgroups of CCA, for which there are still few therapies and the prognosis is unhealthy.

## Supplementary information

Supplementary Figure 1

Supplementary figure 2

Supplementary Figure Legends

Supplementary Table 1

Supplementary Table 2

Supplementary Table 3

## References

[1] Ferlay J (2015). Cancer incidence and mortality worldwide: sources, methods and major patterns in GLOBOCAN 2012. Int. J. Cancer.

[2] Ziogas IA, Tsoulfas G (2017). Evolving role of Sorafenib in the management of hepatocellular carcinoma. World J. Clin. Oncol..

[3] Bhide RS (2010). The antiangiogenic activity in xenograft models of brivanib, a dual inhibitor of vascular endothelial growth factor receptor-2 and fibroblast growth factor receptor-1 kinases. Mol. Cancer Ther..

[4] Park JW (2011). Phase II, open-label study of brivanib as first-line therapy in patients with advanced hepatocellular carcinoma. Clin. Cancer Res..

[5] Johnson PJ (2013). Brivanib versus sorafenib as first-line therapy in patients with unresectable, advanced hepatocellular carcinoma: results from the randomized phase III BRISK-FL study. J. Clin. Oncol..

[6] Llovet JM, Hernandez-Gea V (2014). Hepatocellular carcinoma: reasons for phase III failure and novel perspectives on trial design. Cancer Res..

[7] Lamarca A, Mendiola M, Barriuso J (2016). Hepatocellular carcinoma: exploring the impact of ethnicity on molecular biology. Crit. Rev. Oncol. Hematol..

[8] Hsu C, Shen YC, Cheng AL (2009). Sorafenib for the treatment of hepatocellular carcinoma across geographic regions. Expert Rev. Clin. Pharmacol..

[9] Giovannini C, Bolondi L, Gramantieri L (2016). Targeting Notch3 in hepatocellular carcinoma: molecular mechanisms and therapeutic perspectives. Int. J. Mol. Sci..

[10] Zhang Q (2015). Notch3 functions as a regulator of cell self-renewal by interacting with the beta-catenin pathway in hepatocellular carcinoma. Oncotarget.

[11] Hu L, Xue F, Shao M, Deng A, Wei G (2013). Aberrant expression of Notch3 predicts poor survival for hepatocellular carcinomas. Biosci. Trends.

[12] Giovannini C (2014). Suppression of p53 by Notch3 is mediated by Cyclin G1 and sustained by MDM2 and miR-221 axis in hepatocellular carcinoma. Oncotarget.

[13] Giovannini C (2009). Selective ablation of Notch3 in HCC enhances doxorubicin’s death promoting effect by a p53 dependent mechanism. J. Hepatol..

[14] Giovannini C (2017). Corrigendum: Vidatox 30 CH has tumor activating effect in hepatocellular carcinoma. Sci. Rep..

[15] Giovannini C, Lacchini M, Gramantieri L, Chieco P, Bolondi L (2006). Notch3 intracellular domain accumulates in HepG2 cell line. Anticancer Res..

[16] Gramantieri L (2007). Aberrant Notch3 and Notch4 expression in human hepatocellular carcinoma. Liver Int..

[17] Von Manstein V (2013). Resistance of cancer cells to targeted therapies through the activation of compensating signaling loops. Curr. Signal Transduct. Ther..

[18] Yuan X (2015). Notch signaling: an emerging therapeutic target for cancer treatment. Cancer Lett..

[19] Giovannini C (2012). CDKN1C/P57 is regulated by the Notch target gene Hes1 and induces senescence in human hepatocellular carcinoma. Am. J. Pathol..

[20] Giovannini C (2013). Notch3 inhibition enhances sorafenib cytotoxic efficacy by promoting GSK3b phosphorylation and p21 down-regulation in hepatocellular carcinoma. Oncotarget.

[21] Zhu W (2017). Combination of sorafenib and Valproic acid synergistically induces cell apoptosis and inhibits hepatocellular carcinoma growth via down-regulating Notch3 and pAkt. Am. J. Cancer Res..

[22] Karplus PA (2015). A primer on peroxiredoxin biochemistry. Free Radic. Biol. Med..

[23] Nicolussi A, D’Inzeo S, Capalbo C, Giannini G, Coppa A (2017). The role of peroxiredoxins in cancer. Mol. Clin. Oncol..

[24] Zhou S (2016). PRDX2 protects hepatocellular carcinoma SMMC-7721 cells from oxidative stress. Oncol. Lett..

[25] Liu Z (2016). Silencing PRDX3 inhibits growth and promotes invasion and extracellular matrix degradation in hepatocellular carcinoma cells. J. Proteome Res..

[26] Xu X (2016). The phospholipase A2 activity of peroxiredoxin 6 promotes cancer cell death induced by tumor necrosis factor alpha in hepatocellular carcinoma. Mol. Carcinog..

[27] Guest RV (2016). Notch3 drives development and progression of cholangiocarcinoma. PNAS.

[28] Wu WR (2014). Clinicopathological significance of aberrant Notch receptors in intrahepatic cholangiocarcinoma. Int. J. Clin. Exp. Pathol..

[29] Leontovich AA (2018). NOTCH3 expression is linked to breast cancer seeding and distant metastasis. Breast Cancer Res..

[30] Zhang Z (2010). Notch3 in human breast cancer cell lines regulates osteoblast-cancer cell interactions and osteolytic bone metastasis. Am. J. Pathol..

[31] Yamaguchi N (2008). NOTCH3 signaling pathway plays crucial roles in the proliferation of ErbB2-negative human breast cancer cells. Cancer Res..

[32] Choy L (2017). Constitutive NOTCH3 signaling promotes the growth of basal breast cancers. Cancer Res..

[33] Ma D (2011). Aberrant expression and clinical correlation of Notch signaling molecules in breast cancer of Chinese population. Asia-Pac. J. Clin. Oncol..

[34] Diluvio G (2018). NOTCH3 inactivation increases triple negative breast cancer sensitivity to gefitinib by promoting EGFR tyrosine dephosphorylation and its intracellular arrest. Oncogenesis.

[35] Wang D (2018). IL6 blockade potentiates the anti-tumor effects of gamma-secretase inhibitors in Notch3-expressing breast cancer. Cell Death Diff..

[36] Sansone P (2007). p66Shc/Notch-3 interplay controls self-renewal and hypoxia survival in human stem/progenitor cells of the mammary gland expanded in vitro as mammospheres. Stem Cells.

[37] DeBerardinis RJ, Chandel NS (2016). Fundamentals of cancer metabolism. Sci. Adv..

[38] Vander Heiden MG, DeBerardinis RJ (2017). Understanding the intersections between metabolism and cancer biology. Cell.

[39] Kim HK (2017). Current and upcoming mitochondrial targets for cancer therapy. Semin. Cancer Biol..

[40] Erez A, DeBerardinis RJ (2015). Metabolic dysregulation in monogenic disorders and cancer - finding method in madness. Nat. Rev. Cancer.

[41] Joutel A (2000). The ectodomain of the Notch3 receptor accumulates within the cerebrovasculature of CADASIL patients. J. Clin. Invest..

[42] Radtke F, Wilson A, MacDonald HR (2004). Notch signaling in T- and B-cell development. Curr. Opin. Immunol..

[43] Zhou JX (2016). Association between high levels of Notch3 expression and high invasion and poor overall survival rates in pancreatic ductal adenocarcinoma. Oncol. Rep..

[44] Ye YZ (2013). Notch3 overexpression associates with poor prognosis in human non-small-cell lung cancer. Med. Oncol..

[45] Floter J, Kaymak I, Schulze A (2017). Regulation of metabolic activity by p53. Metabolites.

[46] Burns JS, Manda G (2017). Metabolic pathways of the warburg effect in health and disease: perspectives of choice, chain or chance. Int. J. Mol. Sci..

[47] Anderson NM, Mucka P, Kern JG, Feng H (2018). The emerging role and targetability of the TCA cycle in cancer metabolism. Protein Cell.

[48] Gottschalk S, Anderson N, Hainz C, Eckhardt SG, Serkova NJ (2004). Imatinib (STI571)-mediated changes in glucose metabolism in human leukemia BCR-ABL-positive cells. Clin. Cancer Res..

