# Supplementary figures and images for: Brivanib in combination with Notch3 silencing shows potent activity in tumour models
Source: Br J Cancer. 2019 Feb 15;120(6):601–11. doi: 10.1038/s41416-018-0375-4 (PMC6461893; doi:10.1038/s41416-018-0375-4)

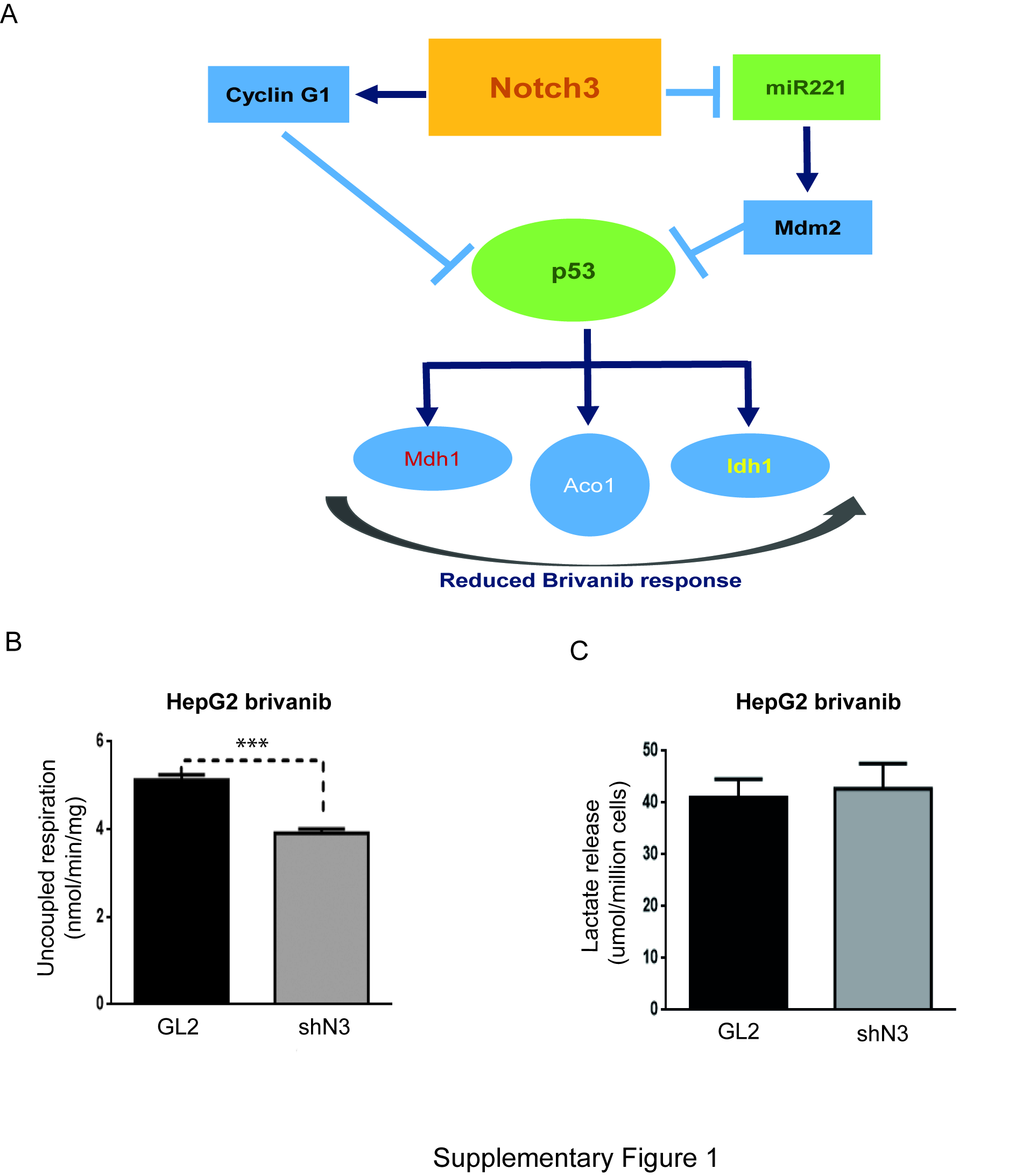

Supplement: Supplementary file 1 — Supplementary Figure 1 [file 41416_2018_375_MOESM1_ESM.tif]

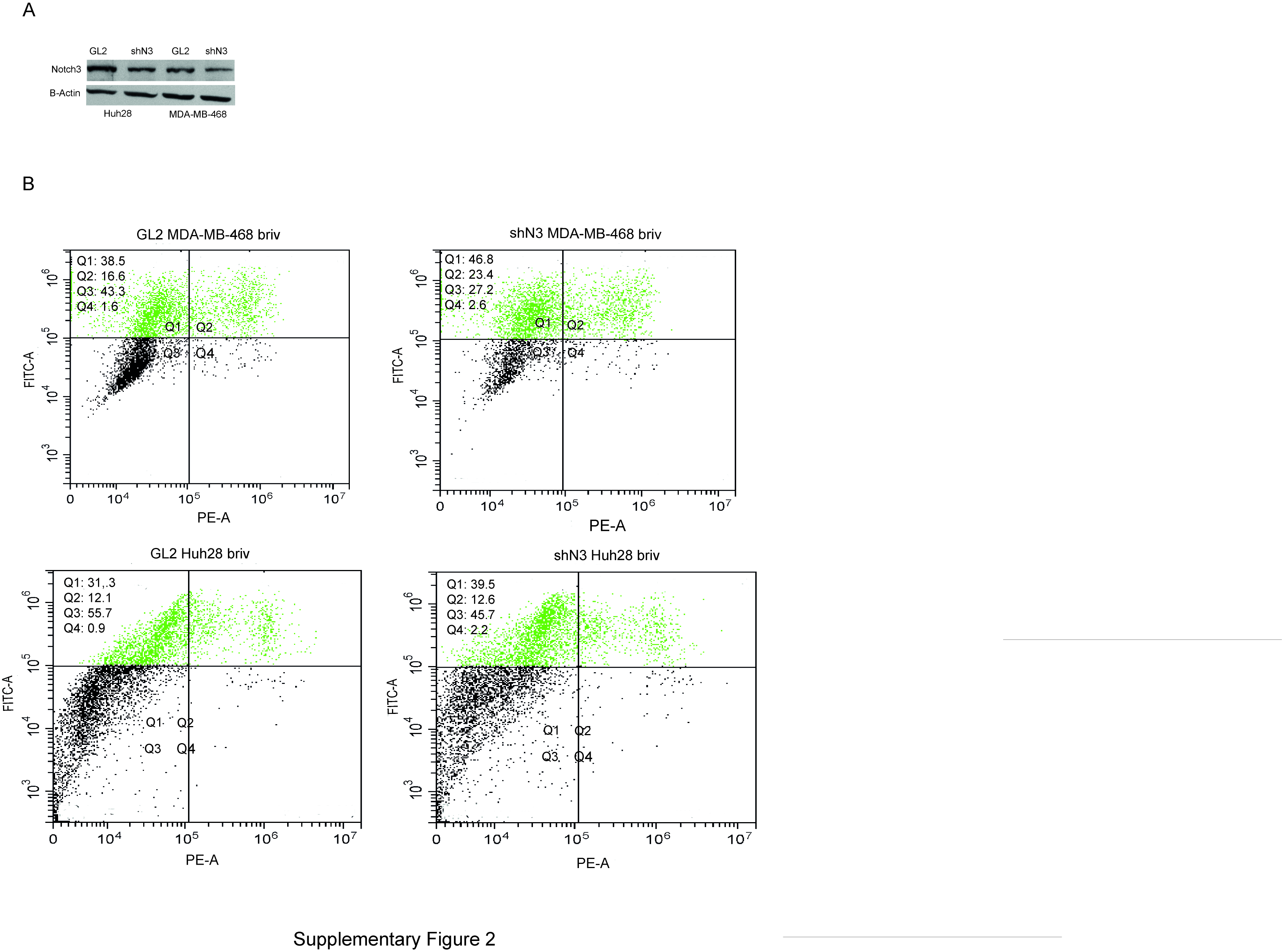

Supplement: Supplementary file 2 — Supplementary figure 2 [file 41416_2018_375_MOESM2_ESM.tif]
