# Supplementary material for: Brivanib in combination with Notch3 silencing shows potent activity in tumour models
Source: Br J Cancer. 2019 Feb 15;120(6):601–11. doi: 10.1038/s41416-018-0375-4 (PMC6461893; doi:10.1038/s41416-018-0375-4)
Supplement: Supplementary file 3 — Supplementary Figure Legends [file 41416_2018_375_MOESM3_ESM.docx]

**SUPPLEMENTAL FIGURE LEGENDS**

**Supplemental Figure 1: Notch3 depletion effects.** A) Relation between Notch3, p53, Mdh1, and Idh1. B) Respiration rate, expressed as nanomole of O2/min/mg of protein after 72h of brivanib exposure, was represented. C) Lactate release analysis performed in control (GL2) and Notch3 silenced cells (shN3) treated for 72h with brivanib. Results are the mean of three independent experiments (+/- S.E.). ***, p<0.001 values by two tailed student’s t test.

**Supplementary Figure 2: Notch3 silencing increases the apoptosis inducing effect of brivanib in cholangiocarcinoma and in breast cancer cells.**

A) Notch3 protein expression evaluated by western blotting in Huh28 and MDA-MB-468 negative control (GL2) and Notch3-silenced cells (shN3). B) After treatment with 60 μM brivanib for 72 h, Huh28 and MDA-MB-468 cells were labeled with annexin V-FITC and propidium iodide. The distribution pattern of live and apoptotic cells was determined by FACS analysis.
