# Supplementary material for: Brivanib in combination with Notch3 silencing shows potent activity in tumour models
Source: Br J Cancer. 2019 Feb 15;120(6):601–11. doi: 10.1038/s41416-018-0375-4 (PMC6461893; doi:10.1038/s41416-018-0375-4)
Supplement: Supplementary file 4 — Supplementary Table 1 [file 41416_2018_375_MOESM4_ESM.docx]

**Supplementary Table 1.** Differentially represented proteins in HepG2-shN3 cells in comparison with HepG2-GL2 treated with brivanib, as deriving from combined 2D-DIGE/nLC-ESI-LIT-MS/MS analysis. See the experimental section for protein extraction and proteomic analysis. Score, number of identified peptide matches/sequences, sequence coverage (%) and emPAI values result from Mascot database searching of mass spectrometry data.

| **SPOT** | **Protein accession** | **Protein description** | **Score** | **MW th (Da)** | **∑ Peptide matches** | **∑**  **Peptide sequences** | **cover (%)** | **pI th** | **emPAI** | **Av. ratio shN3Briv *vs***  **Briv** | **T-test** |
| --- | --- | --- | --- | --- | --- | --- | --- | --- | --- | --- | --- |
| 704 | sp\|O60701\|UGDH_HUMAN | UDP-glucose 6-dehydrogenase | 163 | 55674 | 5 | 4 | **10.3** | 6.73 | 0.25 | -1.55 | 0.0085 |
| 895 | sp\|P13639\|EF2_HUMAN | Elongation factor 2 | 415 | 96246 | 12 | 9 | **12.2** | 6.41 | 0.4 | 1.77 | 0.013 |
| 900 | sp\|Q14697\|GANAB_HUMAN | Neutral alpha-glucosidase AB | 192 | 107263 | 3 | 3 | **5.6** | 5.74 | 0.12 | 1.89 | 0.0023 |
| 906 | sp\|P55072\|TERA_HUMAN | Transitional endoplasmic reticulum ATPase | 180 | 89950 | 4 | 4 | **6.1** | 5.14 | 0.18 | 1.59 | 0.012 |
| 922 | sp\|Q14697\|GANAB_HUMAN | Neutral alpha-glucosidase AB | 217 | 107263 | 7 | 5 | **7.4** | 5.74 | 0.21 | 1.89 | 0.00075 |
| 954 | sp\|P21399\|ACOC_HUMAN | Cytoplasmic aconitate hydratase | 244 | 98850 | 7 | 6 | **7.3** | 6.23 | 0.23 | -1.51 | 0.0063 |
| 1226 | sp\|P52888\|THOP1_HUMAN | Thimet oligopeptidase | 255 | 79702 | 6 | 6 | **10.7** | 5.72 | 0.31 | -2.22 | 7.7e-006 |
| 1233 | sp\|P52888\|THOP1_HUMAN | Thimet oligopeptidase | 412 | 79702 | 9 | 9 | **13.8** | 5.72 | 0.51 | -1.64 | 0.0099 |
| 1289 | sp\|P41250\|SYG_HUMAN | Glycine--tRNA ligase | 471 | 83854 | 15 | 11 | **14.1** | 6.61 | 0.48 | -1.77 | 0.00094 |
| 1437 | sp\|P20700\|LMNB1_HUMAN | Lamin-B1 | 494 | 66653 | 10 | 10 | **17.2** | 5.11 | 0.63 | 4.82 | 0.010 |
| 1455 | sp\|P08107\|HSP71_HUMAN | Heat shock 70 kDa protein 1A/1B | 284 | 70294 | 5 | 5 | **9.2** | 5.48 | 0.2 | 2.71 | 0.0011 |
| 1461 | sp\|P08107\|HSP71_HUMAN | Heat shock 70 kDa protein 1A/1B | 484 | 70294 | 9 | 9 | **16.7** | 5.48 | 0.61 | 2.84 | 5.1e-005 |
| 1503 | sp\|Q03252\|LMNB2_HUMAN | Lamin-B2 | 652 | 67762 | 9 | 9 | **18.0** | 5.29 | 0.51 | 2.06 | 0.0078 |
| 1517 | sp\|P49748\|ACADV_HUMAN | Very long-chain specific acyl-CoA dehydrogenase, mitochondrial | 313 | 70745 | 6 | 6 | **9.3** | 8.92 | 0.33 | -1.63 | 0.0018 |
| 1561 | sp\|O95394\|AGM1_HUMAN | Phosphoacetylglucosamine mutase | 197 | 60270 | 4 | 4 | **7.7** | 5.84 | 0.19 | -1.69 | 0.00034 |
| 1596 | sp\|P61978\|HNRPK_HUMAN | Heterogeneous nuclear ribonucleoprotein K | 211 | 51230 | 5 | 4 | **12.7** | 5.39 | 0.32 | 1.82 | 0.0030 |
| 1656 | sp\|P10809\|CH60_HUMAN | 60 kDa heat shock protein, mitochondrial | 436 | 61187 | 9 | 8 | **15.7** | 5.7 | 0.51 | 1.59 | 0.035 |
| 1797 | sp\|P30101\|PDIA3_HUMAN | Protein disulfide-isomerase A3 | 456 | 57146 | 12 | 9 | **22.2** | 5.98 | 0.7 | 2.27 | 0.00099 |
| 1799 | sp\|P30101\|PDIA3_HUMAN | Protein disulfide-isomerase A3 | 492 | 57146 | 12 | 10 | **23.4** | 5.98 | 0.69 | 2.46 | 0.00013 |
| 1851 | sp\|P27797\|CALR_HUMAN | Calreticulin | 187 | 48283 | 4 | 4 | **12.2** | 4.29 | 0.54 | 1.75 | 0.0040 |
| 1894 | sp\|P10809\|CH60_HUMAN | 60 kDa heat shock protein, mitochondrial | 245 | 61187 | 5 | 5 | **8.4** | 5.7 | 0.34 | 1.73 | 0.045 |
| 1903 | sp\|P55209\|NP1L1_HUMAN | Nucleosome assembly protein 1-like 1 | 191 | 45631 | 4 | 4 | **10.0** | 4.36 | 1.17 | 1.66 | 0.0015 |
| 1975 | sp\|P06576\|ATPB_HUMAN | ATP synthase subunit beta, mitochondrial | 133 | 56525 | 2 | 2 | **5.1** | 5.26 | 0.21 | 2.38 | 0.0026 |
| 2040 | sp\|P50395\|GDIB_HUMAN | Rab GDP dissociation inhibitor beta | 812 | 51087 | 19 | 15 | **42.2** | 6.11 | 2.05 | -1.66 | 0.011 |
| 2145 | sp\|O75821\|EIF3G_HUMAN | Eukaryotic translation initiation factor 3 subunit G | 137 | 35874 | 3 | 3 | **11.3** | 5.87 | 0.35 | -1.83 | 0.0035 |
| 2166 | sp\|P23526\|SAHH_HUMAN | Adenosylhomocysteinase | 224 | 48255 | 4 | 4 | **11.3** | 5.92 | 0.35 | -6.80 | 3.2e-005 |
| 2178 | sp\|P63261\|ACTG_HUMAN | Actin, cytoplasmic 2 | 267 | 42108 | 8 | 6 | **16.8** | 5.31 | 0.98 | 1.60 | 0.0094 |
| 2231 | sp\|O75821\| EIF3G_HUMAN | Eukaryotic translation initiation factor 3 subunit G (Fragment) | 211 | 29550 | 4 | 4 | **14.5** | 5.31 | 0.62 | -2.15 | 0.00055 |
| 2250 | sp\|O75874\|IDHC_HUMAN | Isocitrate dehydrogenase [NADP] cytoplasmic | 154 | 46915 | 3 | 3 | **9.9** | 6.53 | 0.26 | -2.39 | 0.00012 |
| 2272 | sp\|Q9BQA1\|MEP50_HUMAN | Methylosome protein 50 | 130 | 37442 | 2 | 2 | **7.9** | 5.03 | 0.21 | 2.35 | 0.0012 |
| 2282 | sp\|P63261\|ACTG_HUMAN | Actin, cytoplasmic 2 | 214 | 42108 | 6 | 5 | **14.1** | 5.31 | 0.67 | 1.61 | 0.045 |
| 2540 | sp\|P53004\|BIEA_HUMAN | Biliverdin reductase A | 152 | 33692 | 3 | 3 | **13.2** | 6.06 | 0.37 | -1.97 | 0.00061 |
| 2587 | sp\|P37837\|TALDO_HUMAN | Transaldolase | 220 | 37688 | 4 | 4 | **11.9** | 6.36 | 0.46 | -1.65 | 0.0023 |
| 2610 | sp\|O60218\|AK1BA_HUMAN | Aldo-keto reductase family 1 member B10 | 274 | 36225 | 6 | 6 | **16.1** | 7.67 | 0.64 | -2.90 | 0.00039 |
| 2679 | sp\|P40925\|MDHC_HUMAN | Malate dehydrogenase, cytoplasmic | 179 | 36631 | 3 | 3 | **12.9** | 6.91 | 0.54 | -3.81 | 0.00020 |
| 2689 | sp\|O75822\|EIF3J_HUMAN | Eukaryotic translation initiation factor 3 subunit J | 107 | 29159 | 2 | 2 | **9.7** | 4.72 | 0.28 | 2.49 | 2.6e-005 |
| 2727 | sp\|P50224\|ST1A3_HUMAN | Sulfotransferase 1A3/1A4 | 142 | 34288 | 3 | 3 | **12.2** | 5.68 | 0.37 | -1.66 | 0.042 |
| 2798 | sp\|Q06520\|ST2A1_HUMAN | Bile salt sulfotransferase | 382 | 33929 | 10 | 7 | **31.6** | 5.71 | 1.32 | -1.56 | 8.3e-005 |
| 2916 | sp\|Q13011\|ECH1_HUMAN | Delta(3,5)-Delta(2,4)-dienoyl-CoA isomerase, mitochondrial | 171 | 36136 | 10 | 10 | **43.6** | 8.16 | 0.49 | -1.96 | 0.00032 |
| 2918 | sp\|P50225\|ST1A1_HUMAN | Sulfotransferase 1A1 | 393 | 34289 | 12 | 9 | **33.2** | 6.16 | 1.55 | -2.32 | 1.5e-005 |
| 2935 | sp\|P61289\|PSME3_HUMAN | Proteasome activator complex subunit 3 | 265 | 29602 | 5 | 4 | **19.7** | 5.69 | 0.78 | -1.64 | 0.0012 |
| 3056 | sp\|O43399\|TPD54_HUMAN | Tumor protein D54 | 465 | 22281 | 10 | 9 | **51.5** | 5.26 | 2.55 | -1.70 | 0.043 |
| 3152 | sp\|O43399\|TPD54_HUMAN | Tumor protein D54 | 250 | 22281 | 4 | 4 | **26.7** | 5.26 | 1.02 | -1.75 | 0.013 |
| 3227 | sp\|Q13162\|PRDX4_HUMAN | Peroxiredoxin-4 | 159 | 30749 | 3 | 3 | **12.2** | 5.86 | 0.46 | 1.63 | 0.014 |
| 3247 | sp\|P30041\|PRDX6_HUMAN | Peroxiredoxin-6 | 475 | 25133 | 22 | 9 | **33.0** | 6.0 | 2.57 | 3.59 | 0.00057 |
| 3468 | sp\|P30048\|PRDX3_HUMAN | Thioredoxin-dependent peroxide reductase, mitochondrial | 174 | 28017 | 6 | 3 | **15.2** | 7.67 | 0.89 | 1.91 | 0.0035 |
| 3543 | sp\|P30085\|KCY_HUMAN | UMP-CMP kinase | 230 | 22436 | 4 | 4 | **23.5** | 5.44 | 0.72 | -1.71 | 0.021 |
| 3575 | sp\|P32119\|PRDX2_HUMAN | Peroxiredoxin-2 | 378 | 22049 | 10 | 7 | **32.8** | 5.66 | 3.03 | -1.55 | 0.031 |
| 3733 | sp\|O43169\|CYB5B_HUMAN | Cytochrome b5 type B | 121 | 15878 | 2 | 2 | **22.1** | 5.74 | 0.55 | 1.69 | 0.0060 |
| 4128 | sp\|P62760\|VISL1_HUMAN | Visinin-like protein 1 | 227 | 22299 | 4 | 4 | **26.2** | 5.01 | 1.4 | 1.78 | 0.034 |
