# Supplementary material for: Brivanib in combination with Notch3 silencing shows potent activity in tumour models
Source: Br J Cancer. 2019 Feb 15;120(6):601–11. doi: 10.1038/s41416-018-0375-4 (PMC6461893; doi:10.1038/s41416-018-0375-4)
Supplement: Supplementary file 6 — Supplementary Table 3 [file 41416_2018_375_MOESM6_ESM.docx]

**Supplementary Table 3.** **Biological process clustering of the differentially represented proteins identified in HepG2-shN3 cells.**

The table illustrates the distribution according to corresponding biological processes of over-and down-represented proteins in HepG2-shN3 compared to HepG2-GL2 cells after preventive treatment with brivanib for 72 h. Categorization was based on information provided by functional annotation tools of DAVID software v. 6.8. Only categories (GOTERM_BP_DIRECT) showing *P*-value ≤ 0.01 are reported.

| **GO category - Biological Process** | **GO id** | **N. of genes** | **%** | ***P*-Value** | **Benjamini** |
| --- | --- | --- | --- | --- | --- |
| Cell redox homeostasis | GO:0045454 | \| 5 \| \| --- \| | 11.9 | 3.0E-5 | 1.0E-2 |
| Oxidation-reduction process | GO:0055114 | \| 9 \| \| --- \| | 21.4 | 5.3E-5 | 9.0E-3 |
| Steroid metabolic process | GO:0008202 | \| 4 \| \| --- \| | 9.5 | 1.3E-4 | 1.5E-2 |
| Sulfation | GO:0051923 | \| 3 \| \| --- \| | 7.1 | 2.9E-4 | 2.4E-2 |
| 3'-Phosphoadenosine 5'-phosphosulfate metabolic process | GO:0050427 | \| 3 \| \| --- \| | 7.1 | 5.4E-4 | 3.6E-2 |
| Carbohydrate metabolic process | GO:0005975 | \| 5 \| \| --- \| | 11.9 | 6.9E-4 | 3.8E-2 |
| Hydrogen peroxide catabolic process | GO:0042744 | \| 3 \| \| --- \| | 7.1 | 9.7E-4 | 4.6E-2 |
| Tricarboxylic acid cycle | GO:0045454 | \| 3 \| \| --- \| | 7.1 | 2.1E-3 | 8.3E-2 |
| ATP metabolic process | GO:0046034 | \| 3 \| \| --- \| | 7.1 | 2.5E-3 | 9.0E-2 |
| Cell-cell adhesion | GO:0098609 | \| 5 \| \| --- \| | 11.9 | 3.5E-3 | 1.1E-1 |
| Response to reactive oxygen species | GO:0000302 | \| 3 \| \| --- \| | 7.1 | 3.7E-3 | 1.1E-1 |
| response to hydrogen peroxide | GO:0042542 | \| 3 \| \| --- \| | 7.1 | 6.2E-3 | 1.6E-1 |
| Cellular response to oxidative stress | GO:0034599 | \| 3 \| \| --- \| | 7.1 | 9.7E-3 | 2.2E-1 |
